# Promoting electrocatalytic overall water splitting by sulfur incorporation into CoFe-(oxy)hydroxide†

**DOI:** 10.1039/d1na00486g

**Published:** 2021-09-09

**Authors:** Chiho Kim, Seunghun Lee, Seong Hyun Kim, Ilyeong Kwon, Jaehan Park, Shinho Kim, Jae-ho Lee, Yoo Sei Park, Yangdo Kim

**Affiliations:** Department of Materials Science and Engineering, Pusan National University Busan 46241 Republic of Korea; BK21 four, Innovative Graduate Education Program for Global High-tech Materials & Parts, Pusan National University Busan 46241 Republic of Korea; Department of Materials Science and Engineering, Hongik University Seoul 04066 Republic of Korea qkrdbtp@pusan.ac.kr yangdo@pusan.ac.kr

## Abstract

The design and fabrication of highly cost-effective electrocatalysts with high activity, and stability to enhance the hydrogen evolution reaction (HER) and oxygen evolution reaction (OER) has been considered to be one of the most promising approaches toward overall water splitting. In this study, sulfur-incorporated cobalt–iron (oxy)hydroxide (S-(Co,Fe)OOH) nanosheets were directly grown on commercial iron foam *via* galvanic corrosion and hydrothermal methods. The incorporation of sulfur into (Co,Fe)OOH results in superior catalytic performance and high stability in both the HER and OER conducted in 1 M KOH. The incorporation of sulfur enhanced the electrocatalytic activity by modifying the electronic structure and chemical states of (Co,Fe)OOH. An alkaline water electrolyzer for overall water splitting was fabricated using a two-electrode configuration utilizing the S-(Co,Fe)OOH bifunctional electrocatalyst in both the HER and OER. The fabricated electrolyzer outperformed a precious metal-based electrolyzer using Pt/C as the HER electrocatalyst and IrO_2_ as the OER electrocatalyst, which are the benchmark catalysts. This electrolyzer provides a lower potential of 1.641 V at 10 mA cm^−2^ and maintains 98.4% of its performance after 50 h of durability testing. In addition, the S-(Co,Fe)OOH-based electrolyzer successfully generated hydrogen under natural illumination upon its combination with a commercial silicon solar cell and exhibited a solar to hydrogen (STH) efficiency of up to 13.0%. This study shows that S-(Co,Fe)OOH is a promising candidate for application in the future renewable energy industry due to its high cost-effectiveness, activity, and stability during overall water splitting. In addition, the combination of a commercial silicon solar cell with an alkaline water electrolyzer has great potential for the production of hydrogen.

## Abstract 2

The incorporation of sulfur into (Co,Fe)-oxyhydroxide induced the change of the electronic structure and enhanced overall water splitting.

## Graphical abstract

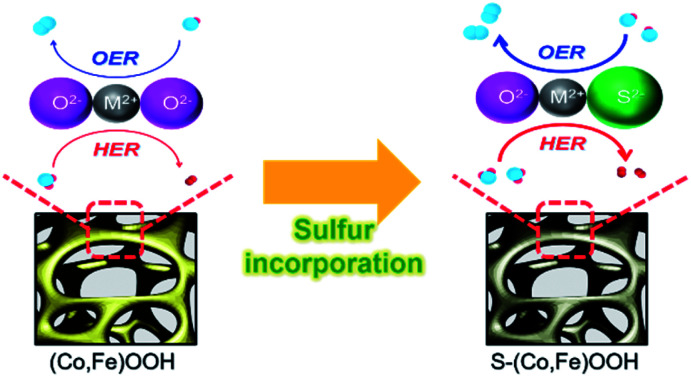

## 1. Introduction

Hydrogen energy has attracted a lot of attention as a next-generation renewable fuel with high density and infinite resources.^1^ In particular, electrochemical alkaline water splitting is considered to be an effective and clean method for producing hydrogen energy.^2–4^ Alkaline water splitting is composed of the hydrogen evolution reaction (HER; 2H_2_O + 2e^−^ → 2OH^−^ + H_2_) and the oxygen evolution reaction (OER; 4OH^−^ → 2H_2_O + 4e^−^ + O_2_).^5^ However, hydrogen production using these two reactions has one fatal obstacle the reduction in the hydrogen production efficiency observed due to the slow kinetics and complexity of each reaction.^6–9^ Therefore, to achieve highly efficient water splitting, it is necessary to develop highly active OER/HER electrocatalysts.

In general, precious metal-based electrocatalysts are considered as benchmark electrocatalysts used for the HER (Pt-based) and OER (IrO_2_-based).^10,11^ However, the high price, scarcity, and poor stability of these precious metals have restricted their use in large-scale applications.^6,12–15^ To overcome these problems, many strategies have been developed using cost-effective and earth-abundant non-precious metals exhibiting high electrocatalytic activity.^16–21^ For this purpose, several types of earth-abundant transition metal-based electrocatalysts, such as Fe, Co, Ni, Cu, and Mn, have been extensively investigated. These include transition metal oxides,^22–24^ phosphides,^25–27^ sulfides,^28–30^ selenides,^31–33^ borides,^34^ and hydroxides.^35–37^ In particular, transition metal-based hydroxides (*i.e.*, hydroxides, layered double hydroxides, and (oxy)hydroxides) have been considered promising candidates as bifunctional electrocatalysts because of their excellent catalytic activity and stability.^38–43^ The development of highly active and cost-effective transition metal hydroxide-based HER and OER bifunctional electrocatalysts for water splitting offers a cost benefit to hydrogen production and the advantage of simplifying the device manufacturing process.^44^ In addition, considering that most first-row transition metals are not stable under acidic conditions, it is essential to develop highly efficient bifunctional electrocatalysts that operate in an integrated alkaline environment for overall water splitting.^45,46^ The high catalytic activity of transition metal-based hydroxides toward the OER has already been demonstrated in many studies. However, they exhibit relatively low catalytic activity in the HER due to their inherently low electrical conductivity.^47–52^ Furthermore, polymeric binders such as Nafion and polytetrafluoroethylene (PTFE) are used to form a cohesive catalyst layer, but degrade the electrode conductivity and performance.^53,54^ Thus, the design of highly active transition metal-based hydroxides for the HER and OER with high electrical conductivity is an important challenge for achieving high efficiency in overall water splitting. One way to improve the catalytic activity of these transition metal-based hydroxides is anion regulation. In particular, incorporating an anion with a relatively low electronegativity in addition to lattice oxygen modifies the adsorption energy between the electrocatalyst and reactant to improve the water splitting process.^55–58^

In this study, we report a bifunctional electrocatalyst using sulfur incorporated cobalt–iron (oxy)hydroxide (S-(Co,Fe)OOH) as both the anode and cathode in alkaline overall water splitting. (Co,Fe)OOH was directly synthesized on iron foam using corrosion engineering and sulfur was then incorporated into (Co,Fe)OOH using a simple hydrothermal method. S-(Co,Fe)OOH exhibits enhanced catalytic activity in both the HER and OER in an alkaline electrolyte when compared to (Co,Fe)OOH due to the effect of sulfur incorporation. An alkaline water electrolyzer, fabricated using S-(Co,Fe)OOH as both the cathode and anode, provides better overall water splitting performance than a precious-metal-based water electrolyzer constructed using Pt/C and IrO_2_ as the cathode and anode.

## 2. Experimental

### 2.1. Synthesis of (Co,Fe)OOH

(Co,Fe)OOH was grown directly on commercial iron foam (IF) using a galvanic corrosion reaction. Iron foam was prepared with a size of 2 × 3 cm^2^ and treated with 1 M HCl solution for 10 min to remove the surface oxide layer. Subsequently, the iron foam was washed immediately with acetone, ethanol, and deionized water under ultrasonication for 10 min. A 3 mM solution of CoCl_2_ (70 mL) was prepared at room temperature. The treated and washed iron foam was immersed in the solution for 2 h at 60 °C with stirring (80 rpm). After the galvanic process, the (Co,Fe)OOH on iron foam was thoroughly rinsed with deionized water and then placed in a convection oven at 70 °C to dry. This sample was labeled as (Co,Fe)OOH.

### 2.2. Synthesis of S-(Co,Fe)OOH

S-(Co,Fe)OOH was prepared using a hydrothermal process from the as-synthesized (Co,Fe)OOH and sodium sulfide. Na_2_S·9H_2_O (1 g) was dissolved in 40 mL of deionized water and the resulting solution was transferred to a 50 mL Teflon-lined stainless-steel autoclave. The as-synthesized (Co,Fe)OOH was immersed in the solution and heated at 100 °C for 12 h. After the reaction was complete, the autoclave was cooled to room temperature and the as-obtained S-(Co,Fe)OOH was washed several times using ethanol and deionized water, and then dried. This sample was labeled as S-(Co,Fe)OOH.

### 2.3. Characterization

X-ray diffraction patterns were measured on an X-ray diffractometer (XRD, UltimaIV, Rigaku) using a Cu-Kα radiation source over the 2*θ* range of 20°–90°. The surface morphology and composition of the samples were determined using field-emission scanning electron microscopy (FE-SEM, CZ/MIRAI LMH, TESCAN). X-ray photoelectron spectroscopy (XPS) was performed to confirm the elemental composition and oxidation states of the various elements using a K-Alpha spectrometer (AXIS SUPRA^+^, KRATOS Analytical). High-resolution transmission electron microscopy (HR-TEM), elemental distribution spectroscopy (EDS), and selected area electron diffraction (SAED) were performed on a TALOS F200X (Thermo Fisher Scientific, USA) instrument.

### 2.4. Electrochemical characterization

Electrochemical measurements were carried out on a potentiostat (Parstat 2273, Princeton Applied Research) using 1 M KOH as the electrolyte in a three-electrode cell system at room temperature. The as-synthesized (Co,Fe)OOH and S-(Co,Fe)OOH samples were used as the working electrode (1 × 1 cm^2^) and Hg/HgO (1 M KOH) was used as the reference electrode. The counter electrode was Pt mesh for the OER and a graphite rod for the HER, respectively. The HER and OER catalytic activities were evaluated using linear sweep voltammetry (LSV) at a scan rate of 1 mV s^−1^. The Tafel slopes were determined from the corresponding polarization curves. The electrochemical surface area (ECSA) was measured using cyclic voltammetry (CV) at different scanning rates (10–160 mV s^−1^) in the non-faradaic region using 1 M KOH to obtain the double-layer capacitance (*C*_dl_). The ECSA was calculated using eqn (1):

1 ECSA = *C*_dl_/*C*_s_

where *C*_s_ is the capacitance of an atomically smooth planar metal surface, which has a value of 40 μF cm^−2^.^59^ The CV was analyzed *versus* the open circuit potential (OCP) after all of the samples were held in the 1 M KOH electrolyte for 30 min. Electrochemical impedance spectroscopy (EIS) was conducted over the frequency range of 200 kHz to 10 Hz with an amplitude of 10.0 mV; the applied overpotential was −0.25 V_RHE_ for the HER and +1.53 V_RHE_ for the OER. The overall water splitting was performed using a two-electrode system, in which both the cathode and the anode were the same sample of S-(Co,Fe)OOH. Benchmark precious metal electrocatalysts (Pt/C and IrO_2_) were prepared for comparison. The ink solution was prepared using 20 mg of Pt/C and IrO_2_ powder, 5 wt% Nafion solution (100 μL) and ethanol (900 μL). The as-prepared ink solution was dispersed *via* ultrasonication for 15 min to form a homogeneous catalyst ink. A droplet of the ink solution was transferred onto the iron foam surface (1 × 1 cm^2^). The loading mass of Pt/C and IrO_2_ was ∼3 mg cm^−2^. Stability tests were performed at a constant current density of −100 mA cm^−2^ (HER) and +100 mA cm^−2^ for 50 h. All of the reported potentials were converted to the reversible hydrogen electrode (RHE) based on the Nernst equation (*V*_RHE_ (V) *vs.* RHE) and all of the electrochemical data were *iR*-corrected.

## 3. Results and discussion

**Fig. 1:**
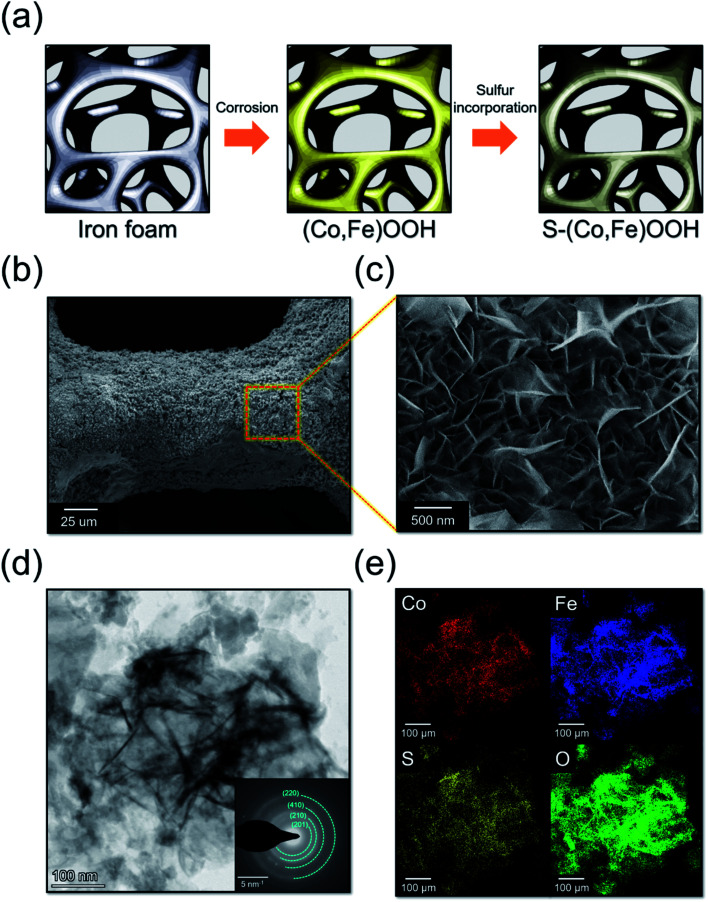
(a) Schematic representation of (Co,Fe)OOH and S-(Co,Fe)OOH. (b) Low- and (c) high-magnification scanning electron microscopy (SEM) images obtained for S-(Co,Fe)OOH. (d) High-resolution transmission electron microscopy (HR-TEM) image of S-(Co,Fe)OOH with its corresponding SAED ring patterns. (e) TEM-EDS mapping images of S-(Co,Fe)OOH.

The (Co,Fe)OOH electrocatalyst was synthesized using galvanic corrosion and oxidation reactions, as shown in Fig. 1(a). Fe was oxidized to Fe^2+^*via* the galvanic coupling of iron foam and Co ions in the CoCl_2_ solution. As seen in the revised Pourbaix diagram of the Co–H_2_O system, Co(OH)_2_ was formed at pH 6 and a high temperature.^60^ The dissolved oxygen can oxidize Fe^2+^ to Fe(OH)_3_ under the same pH conditions.^61^ Coprecipitation of Co(OH)_2_ and Fe(OH)_3_ forms (Co, Fe)OOH on the iron foam surface after drying at a high temperature. The chemical reaction for the synthesis of (Co, Fe)OOH can be described using eqn (2)–(5):^62^

2 Fe + Co^2+^ → Fe^2+^ + Co

3 Co^2+^ + 2H_2_O → Co(OH)_2_ + 2H^+^

4 O_2_ + 2H_2_O + 4e^−^ → 4OH^−^

5 2Fe^2+^ + 4OH^−^ → 2Fe(OH)_2_

6 4Fe(OH)_2_ + O_2_ → 4FeOOH + 2H_2_O

The S-(Co,Fe)OOH electrocatalyst, which is a binder-free electrode, was synthesized using a hydrothermal method from (Co,Fe)OOH and sodium sulfide. Sulfur was incorporated into the as-synthesized (Co,Fe)OOH during the hydrothermal procedure.

To optimize the amount of sulfur, S-(Co,Fe)OOH was synthesized using different amounts of Na_2_S, followed by HER and OER tests, as shown in Fig. S1.† It was confirmed through EDS that the sulfur content in S-(Co,Fe)OOH increased as the amount of Na_2_S used for synthesis increased. The sulfur content in S-(Co,Fe)OOH was 1.9 (0.5 g), 3.2 (1.0 g), and 4.2 at% (1.5 g), respectively. Upon increasing the amount of Na_2_S from 0.5 to 1.0 g, the overpotential (at +10 mA cm^−2^) of the OER decreased from 247 to 240 mV. However, with a Na_2_S amount of 1.5 g, the overpotential increased to 251 mV. Upon increasing the amount of Na_2_S from 0.5 to 1.0 g, the overpotential (at −10 mA cm^−2^) of the HER decreased from 201 to 186 mV. However, with a Na_2_S amount of 1.5 g, the overpotential increased to 227 mV. Therefore, S-(Co,Fe)OOH prepared with 1.0 g Na_2_S is considered to yield more optimal intermediate binding energies for the OER and HER.

Fig. S2† shows the X-ray diffraction patterns obtained for iron foam, (Co,Fe)OOH and S-(Co,Fe)OOH. The XRD patterns only show peaks at 44.7°, 65.2°, and 82.5°, which correspond to metallic iron (JCPDS: 98-063-1729). This result may be ascribed to intense background diffraction peaks of the iron substrate. The scanning electron microscopy (SEM) images obtained for (Co,Fe)OOH are presented in Fig. S4,† and show a nanosheet morphology. In addition, the transmission electron microscopy (TEM) images shown in Fig. S3† also indicate that (Co,Fe)OOH exhibits a nanosheet morphology. The SAED ring pattern of (Co,Fe)OOH was indexed to FeOOH (ICSD: 98-015-9970). The EDS mapping images show a uniform distribution of Co, Fe, and O. S-(Co,Fe)OOH exhibits a nanosheet morphology, indicating that the (Co,Fe)OOH nanosheets were well maintained upon the incorporation of sulfur, as shown in Fig. 1(b and c). In addition, the TEM images obtained for S-(Co,Fe)OOH also indicate its nanosheet morphology, as shown in Fig. 1(d). Interestingly, although sulfur was incorporated into (Co,Fe)OOH, the SAED ring patterns were very similar to those of (Co,Fe)OOH. This means that even when sulfur was incorporated into (Co,Fe)OOH, the phase was well maintained. In addition, the EDS mapping results show that sulfur was well distributed in (Co,Fe)OOH, as shown in Fig. 1(e) and S5.† The atomic percentage of sulfur was confirmed to be ∼3.2% using EDS.

**Fig. 2:**
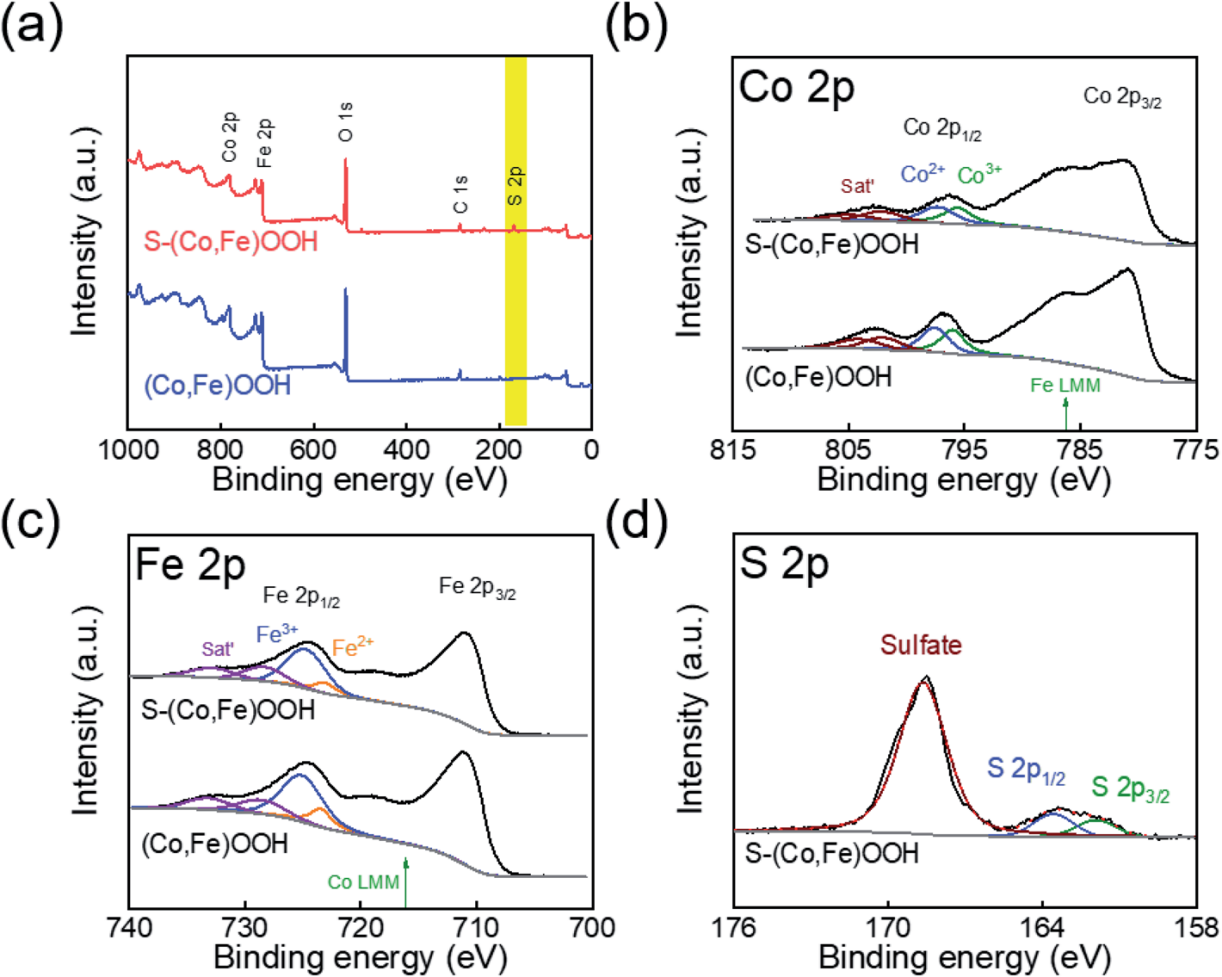
(a) Full X-ray photoelectron spectroscopy (XPS) spectra obtained for (Co,Fe)OOH and S-(Co,Fe)OOH. High resolution XPS spectra obtained for (b) Co 2p, (c) Fe 2p, and (d) S 2p.

XPS was performed to confirm the effect of incorporating sulfur on the chemical states of (Co,Fe)OOH. Fig. 2(a) shows the full XPS survey spectra obtained for (Co,Fe)OOH and S-(Co,Fe)OOH. In the case of (Co,Fe)OOH, it indicates the presence of Co, Fe, and O. S-(Co,Fe)OOH shows the presence of Co, Fe, O, and S. The high-resolution XPS spectra obtained for Co, Fe, and S are shown in Fig. 2(b–d). The chemical states of Co and Fe were analyzed at Co 2p_1/2_ and Fe 2p_1/2_ to avoid the interference of an auger electron.^63,64^ The chemical states of Co were observed to be Co^2+^ and Co^3+^ for both (Co,Fe)OOH and S-(Co,Fe)OOH, as shown in Fig. 2(b). Interestingly, when sulfur was incorporated into (Co,Fe)OOH, a low binding energy shift for Co 2p_1/2_ was observed from 796.85 to 796.49 eV for S-(Co,Fe)OOH. In addition, a low binding energy shift was observed for Fe 2p_1/2_ from 724.66 to 724.40 eV in S-(Co,Fe)OOH, as shown in Fig. 2(c). XPS confirmed that the incorporation of sulfur lowers the binding energies of Co and Fe. These shifts in the binding energies of Co and Fe were caused by the electronegativity and polarization of sulfur because sulfur is an anion with low electronegativity. Sulfur is easier to polarize and can share more dispersive electrons with the adjacent Co and Fe atoms to balance the strong positive field of Co and Fe. Therefore, Co and Fe receive electrons from the incorporated sulfur and as a result, the binding energies of Co and Fe were shifted toward a lower binding energy. The chemical states of sulfur show two major peaks, as shown in Fig. 2(d). The former is oxidized sulfur and the latter corresponds to S 2p_3/2_ and S 2p_1/2_.

**Fig. 3:**
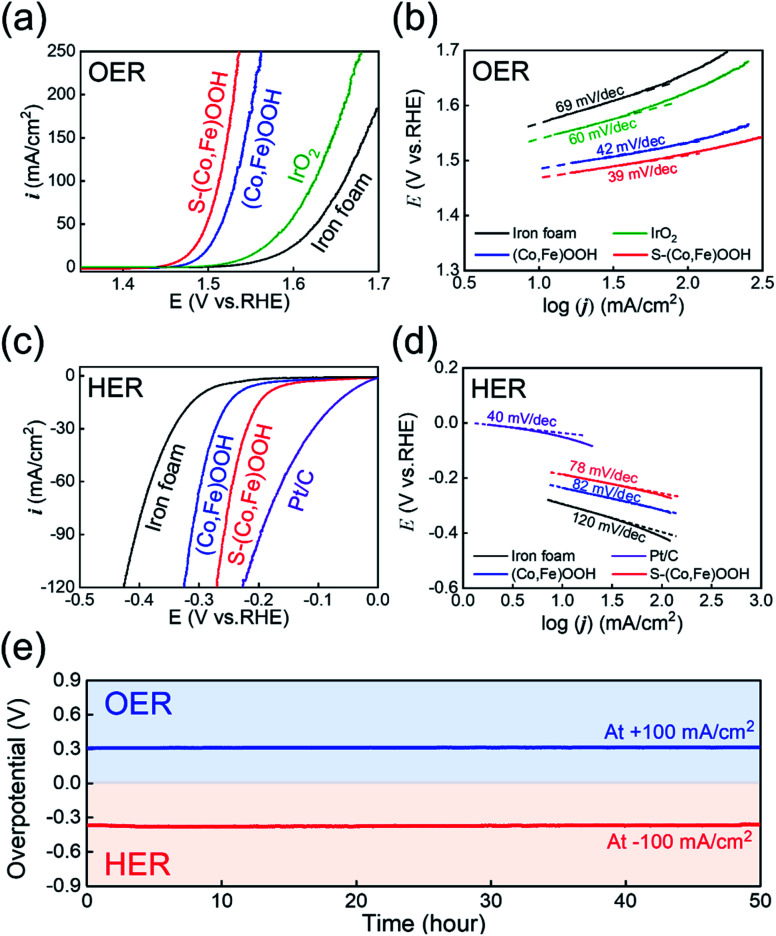
Catalytic activity observed in the OER and HER. (a) Reverse scan polarization curves obtained for the OER. (b) Tafel plots obtained for the OER. (c) Polarization curves obtained for the HER. (d) Tafel plots obtained for the HER. (e) Durability test for the OER and HER carried out at ±100 mA cm^−2^ for 50 h.

Electrochemical tests were performed to confirm the effect of sulfur. The OER electrocatalytic activity was first investigated using linear sweep voltammetry (LSV) performed at an overpotential of 10 mA cm^−2^ in 1 M KOH electrolyte, as shown in Fig. 3(a). S-(Co,Fe)OOH, (Co,Fe)OOH, iron foam, and IrO_2_ were tested under the same conditions in order to compare their OER electrocatalytic activity. Reverse LSV curves were used to avoid any interference from the oxidation reaction.^65,66^ Iron foam exhibited poor OER activity. The precious metal electrocatalyst, IrO_2_, exhibits an overpotential (*η*_OER_) of 311 mV at 10 mA cm^−2^. S-(Co,Fe)OOH exhibits the best OER catalytic activity (*η*_OER_: 240 mV at 10 mA cm^−2^) and the measured overpotential was much lower than those of (Co,Fe)OOH (*η*_OER_: 258 mV at 10 mA cm^−2^) and iron foam (*η*_OER_: 339 mV at 10 mA cm^−2^). These results demonstrate that the OER electrocatalytic activity was enhanced upon the incorporation of sulfur. Tafel plots were obtained to investigate the kinetics of the OER, as shown in Fig. 3(b). The Tafel slope is an important parameter that can be used to determine the mechanism of the OER. The Tafel slopes observed for (Co,Fe)OOH and S-(Co,Fe)OOH were ∼40 mV dec^−1^, indicating that the second electron transfer was the rate-determining step (RDS).^67,68^ The precious metal electrocatalyst, IrO_2_, which is known as a benchmark catalyst, exhibits a Tafel slope of ∼60 mV, indicating that the coverage by the OH^−^ intermediate after the first electron-transfer step was the RDS.^69,70^ From these results, it was confirmed that the intrinsic catalytic activities of (Co,Fe)OOH and S-(Co,Fe)OOH exceed those of precious metals. The HER electrocatalytic activity was investigated by measuring the overpotential at 10 mA cm^−2^ using linear sweep voltammetry (LSV) in a N_2_-purged 1 M KOH electrolyte, as shown in Fig. 3(c). To compare the HER electrocatalytic activity, S-(Co,Fe)OOH, (Co,Fe)OOH, iron foam, and Pt/C were tested under the same conditions. The forward LSV recorded the catalytic activity in the HER. The precious metal electrocatalyst, Pt/C, which is known as a benchmark electrocatalyst, exhibited the lowest overpotential of 49 mV among the electrocatalysts studied. Iron foam exhibited poor HER activity. (Co,Fe)OOH exhibits superior HER catalytic activity with an overpotential of 235 mV at −10 mA cm^−2^. Interestingly, the incorporation of sulfur into (Co,Fe)OOH dramatically enhances the electrocatalytic activity in the HER. S-(Co,Fe)OOH exhibits superior catalytic activity in the HER with an overpotential of 186 mV at −10 mA cm^−2^. Even though S-(Co,Fe)OOH showed enhanced electrocatalytic HER activity, Pt/C was better. However, an interesting phenomenon was observed, in which the current density of S-(Co,Fe)OOH significantly increased upon increasing the voltage when compared to Pt/C. This is because the nanosheet morphology facilitates the mass transfer.^71^Fig. 3(d) shows that the Tafel slope values observed for (Co,Fe)OOH and S-(Co,Fe)OOH in the HER were 82 and 78 mV dec^−1^ (∼90 mV dec^−1^), respectively. However, the precious metal electrocatalyst, Pt/C, which is known as the benchmark catalyst, exhibits a Tafel slope of ∼40 mV dec^−1^, which was lower than those observed for (Co,Fe)OOH and S-(Co,Fe)OOH. The HER involves two theoretical steps. The first step is the Volmer step and the other is the Heyrovsky or Tafel step.^72^ The Tafel slope of Pt/C for the HER was ∼40 mV dec^−1^, which follows the Tafel–Heyrovsky mechanism.^73^ The Tafel slopes observed for (Co,Fe)OOH and S-(Co,Fe)OOH were ∼90 mV dec^−1^, which follows the Volmer–Heyrovsky mechanism.^74,75^ The ECSA, which is another important parameter used to evaluate the electrocatalytic activity, was measured from the double-layer capacitance (*C*_dl_) observed using cyclic voltammetry in the non-faradaic region, as shown in Fig. S7 and S8.† A large ECSA can be considered as an efficient electrocatalyst because it provides abundant active sites for the electrocatalytic reactions to occur.^76,77^ The value of *C*_dl_ increased when sulfur was incorporated into (Co,Fe)OOH, indicating an increase in the active surface area available for the electrochemical reaction. Electrochemical impedance spectroscopy (EIS) was performed to compare the electron charge transfer properties of the different samples, in order to study the OER and HER kinetics, as shown in Fig. S9.† These curves were fitted to an equivalent circuit, where *R*_s_ is the solution resistance and *R*_ct_ is the charge transfer resistance.^78,79^ The OER kinetics were measured at +1.53 V_RHE_ (Fig. S9a†). The *R*_ct_ value of S-(Co,Fe)OOH (0.62 Ω) was much smaller than those observed for (Co,Fe)OOH (0.83 Ω) and iron foam (24.77 Ω), indicating its improved charge transfer properties for the OER. Likewise, EIS measurements used to determine the HER kinetics were performed at −0.25 V_RHE_ (Fig. S9b†). The *R*_ct_ value observed for S-(Co,Fe)OOH (2.06 Ω) was much smaller than those of (Co,Fe)OOH (3.26 Ω) and iron foam (11.31 Ω), indicating its superior charge transfer properties for the HER. The *R*_ct_ value observed for S-(Co,Fe)OOH was smaller when compared to those of the other electrocatalysts studied in both the OER and HER. This result demonstrates that the incorporation of sulfur into (Co,Fe)OOH enhances the kinetics of the OER and HER. The enhanced catalytic activity was attributed to the modification of the electronic structure due to its delocalization characteristics, which indicates that the electrons do not belong to a specific chemical bond, but are likely to exist anywhere in the ring structure, which increases the number of exposed active sites and improves the charge transfer properties. The durability of the S-(Co,Fe)OOH electrocatalyst in the OER and HER was studied by measuring the potential over 50 h at a constant current density of ±100 mA cm^−2^, as shown in Fig. 3(e). A high electrochemical durability of the electrocatalyst is another important property for practical applications. The S-(Co,Fe)OOH electrocatalyst shows excellent durability without any noticeable deterioration during the durability study. One of the main reasons for the excellent electrocatalytic durability is that S-(Co,Fe)OOH has a nanosheet morphology; another reason is that it was grown directly as a binder-free electrocatalyst on the substrate. The gas bubbles generated on the surface during the HER and OER exert enough force to separate from the surface, which causes the destruction of the surface, which depends on the size of the generated bubbles.^80^ The size of the gas bubbles generated on the surface of nanosheets has been reported to be much smaller than those formed on a flat surface;^81^ the effect of this force to destroy the surface was also much smaller. Therefore, the S-(Co,Fe)OOH nanosheet electrocatalyst can maintain high durability, even during the HER and OER. In addition, S-(Co,Fe)OOH grown directly on iron foam exhibits excellent adhesion between the catalyst and substrate, thereby improving the stability of the electrocatalyst. Furthermore, XPS was carried out to confirm the change in the chemical states after the durability tests, as shown in Fig. S10.† Both Co and Fe showed higher oxidation states than those observed before the OER because the OER is an oxidation reaction. The ratio of Co^3+^/Co^2+^ changes from 0.92 to 1.26, and only Fe^3+^ was observed after the OER. The chemical states of sulfur after the OER were well maintained. In particular, the oxidized sulfur species were responsible for the enhanced OER catalytic activity. The residual sulfur modifies the adsorption energy of the reaction intermediates and improves the OER activity.^82^ After the HER, the ratio of Co^3+^/Co^2+^ changes from 0.92 to 0.54 because the HER is a reduction reaction. The chemical states of sulfur also changed. The oxidized sulfur was reduced and the S 2p_3/2_ and S 2p_1/2_ states increased, which also originates from the reduction reaction.

**Fig. 4:**
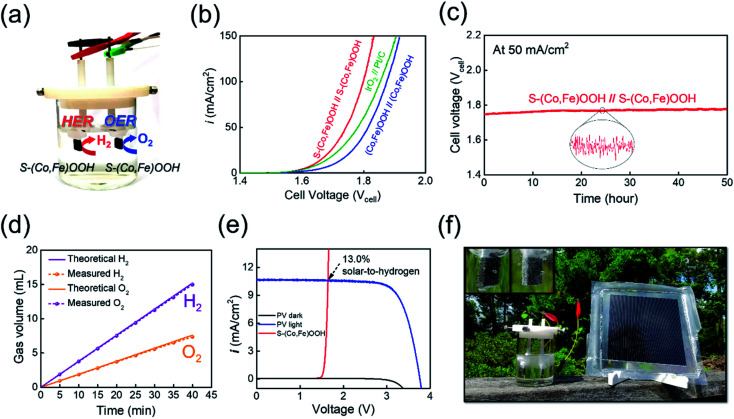
Overall water splitting. (a) A schematic representation of an alkaline water electrolyzer. (b) Polarization curves obtained for the overall water splitting. (c) Durability test carried out at 50 mA cm^−2^ for 50 h. (d) Faradaic efficiency measurements of S-(Co,Fe)OOH at 50 mA cm^−2^. (e) Current density–voltage (*J*–*V*) curve under simulated AM 1.5G 100 mW cm^−2^ illumination for a commercial silicon solar cell combined with the electrolyzer. (f) Photograph of the solar-driven overall water splitting set-up.

For full-cell applications, a two-electrode alkaline electrolyzer was assembled using S-(Co,Fe)OOH as a bifunctional electrocatalyst, as shown in Fig. 4(a). The performance of the electrolyzer constructed using the S-(Co,Fe)OOH electrocatalyst as both the anode and cathode was evaluated for overall water splitting in 1 M KOH solution. A precious metal-based electrolyzer (IrO_2_//Pt/C) and (Co,Fe)OOH-based electrolyzer were also evaluated for comparison under the same conditions. The polarization curves obtained for the two-electrode alkaline electrolyzers investigated in this study are shown in Fig. 4(b). The S-(Co,Fe)OOH-based electrolyzer shows outstanding performance with a cell voltage of 1.641 V (*η*_electrolyzer_: 411 mV) at a current density of 10 mA cm^−2^, which was much better than those observed using the precious metal-based electrolyzer (IrO_2_//Pt/C) (1.656 V) and (Co,Fe)OOH-based electrolyzer (1.705 V). These results prove that the electrocatalytic activity was improved due to the effect of sulfur incorporation. Moreover, the S-(Co,Fe)OOH-based electrolyzer demonstrates better performance in the high current density region. The precious metal-based electrolyzer (IrO_2_//Pt/C) requires 1.85 V to reach a current density of 100 mA cm^−2^, while the S-(Co,Fe)OOH-based electrolyzer needed only 1.79 V. This indicates a greater performance gap than the measured cell voltage obtained at a current density of 10 mA cm^−2^. The performance gap in the high current density region can be attributed to the nanosheet morphology of S-(Co,Fe)OOH. This offers the advantages of a high electrochemical surface area and good mass transfer rate, resulting in the high current density observed at a high potential. The durability of the S-(Co,Fe)OOH-based electrolyzer for the overall water splitting was also evaluated by measuring the voltage over time at a constant current density of 50 mA cm^−2^ for 50 h, as shown in Fig. 4(c). The performance of the S-(Co,Fe)OOH-based electrolyzer was maintained (98.4%) without any noticeable deterioration during the durability test, proving its high activity and stability during the overall water splitting. The faradaic efficiency (FE) of S-(Co,Fe)OOH was measured by collecting the produced O_2_ gas on the anode and H_2_ gas on the cathode at a constant current density of 50 mA cm^−2^, as shown in Fig. 4(d) (and Fig. S11†). The gas volume–time curves showed a high energy conversion with a FE of 98.6%.

Finally, the as-developed S-(Co,Fe)OOH-based electrolyzer was combined with a commercial silicon solar cell to investigate its overall water splitting performance under natural illumination,^83^ as shown in Fig. 4(f). The *J*–*V* curve obtained for the PV device combined with the S-(Co,Fe)OOH-based electrolyzer is shown in Fig. 4(e). In addition, the calculated STH value was 13.0%, demonstrating its high efficiency. The evolution of gas bubbles was clearly observed at both electrodes when the solar-driven water splitting device was driven under natural illumination (Fig. 4(f), inset), showing the successful generation of hydrogen gas. The combination of a PV device and the non-precious metal-based bifunctional electrocatalyst electrolyzer developed in this study demonstrates the potential application of low-cost hydrogen production without the need for artificial currents.

## 4. Conclusions

In summary, a binder-free bifunctional electrocatalyst comprising S-(Co,Fe)OOH has been successfully synthesized using a simple two-step corrosion-hydrothermal method for overall water splitting. The as-synthesized S-(Co,Fe)OOH demonstrated superior catalytic performance in both the HER and OER in 1 M KOH. In addition, S-(Co,Fe)OOH exhibited excellent electrical conductivity, charge transfer properties, kinetics, and a high electrochemical surface area.

These results demonstrate that the incorporation of sulfur enhanced the electrocatalytic activities in both the HER and OER by modifying the electronic structure and chemical states of (Co,Fe)OOH. The bifunctional electrocatalyst electrolyzer constructed using S-(Co,Fe)OOH as both the cathode and anode provided excellent durability and a relatively low potential of 1.641 V at 10 mA cm^−2^, exhibiting outstanding performance compared to a precious metal-based electrolyzer. Moreover, the S-(Co,Fe)OOH-based electrolyzer combined with a commercial silicon solar cell successfully produced hydrogen under natural illumination, exhibiting solar-to-hydrogen (STH) efficiencies of up to 13.0%.

In this study, we present an effective method for the design of a cost-effective, highly active, and stable S-(Co,Fe)OOH electrocatalyst used for the clean and eco-friendly production of hydrogen. In addition, the combination of this developed electrolyzer with a commercial solar cell will provide the possibility of developing a practical solar power system in the future.

## Conflicts of interest

There are no conflicts of interest to declare.

## Supplementary Material

NA-003-D1NA00486G-s001
