# Supplementary material for: Promoting electrocatalytic overall water splitting by sulfur incorporation into CoFe-(oxy)hydroxide
Source: Nanoscale Adv. 2021 Sep 9;3(22):6386–94. doi: 10.1039/d1na00486g (PMC9418770; doi:10.1039/d1na00486g)
Supplement: NA-003-D1NA00486G-s001 [file NA-003-D1NA00486G-s001.pdf]

Electronic Supplementary Material (ESI) for Nanoscale Advances.
This journal is © The Royal Society of Chemistry 2021

Supplementary Information

Promoting electrocatalytic overall water splitting by sulfur incorporation into CoFe(oxy)hydroxide.

Chiho Kim,[a] Seunghun Lee,[a] Seong Hyun Kim,[a] Ilyeong Kwon,[a] Jaehan Park,[a] Shinho Kim,[b] Jae-ho Lee,[c] Yoo Sei Park,*[a] and Yangdo Kim*[a]

AUTHOR ADDRESS.

[a] Department of Materials Science and Engineering, Pusan National University, Busan 46241, Republic of Korea.

[b] BK21 four, Innovative Graduate Education Program for Global High-tech Materials & Parts, Pusan National University, Busan 46241, Republic of Korea.

[c] Department of Materials Science and Engineering, Hongik University, Seoul 04066, Republic of Korea.

* E-mail: qkrdbtp@pusan.ac.kr; yangdo@pusan.ac.kr

KEYWORDS : Overall water splitting reaction; Hydrogen production; Sulfur incorporation; Cobalt–iron (oxy)hydroxide; Bifunctional electrocatalysts.

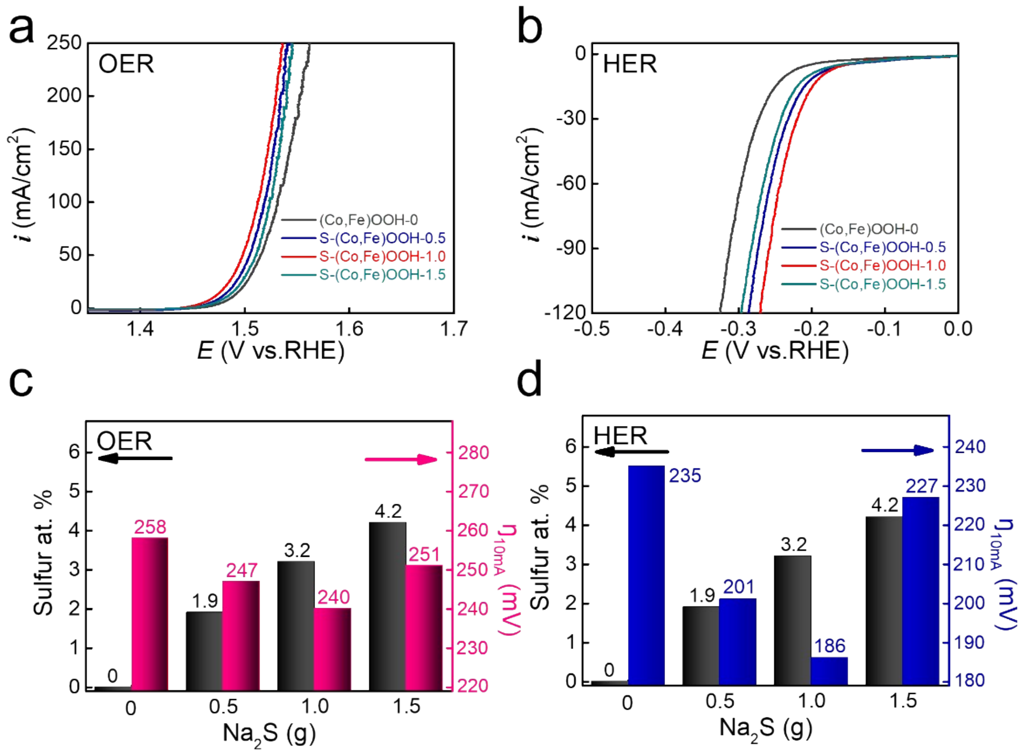

Figure S1. Electrochemical analysis of S-(Co,Fe)OOH prepared with different amounts of Na_2S (0, 0.5, 1.0, and 1.5 g). (a) Polarization curves for OER. (b) Polarization curves for HER. (c) Comparison of sulfur content and overpotential for OER at +10 mA/cm^2. (d) Comparison of sulfur content and overpotential for HER at -10 mA/cm^2.

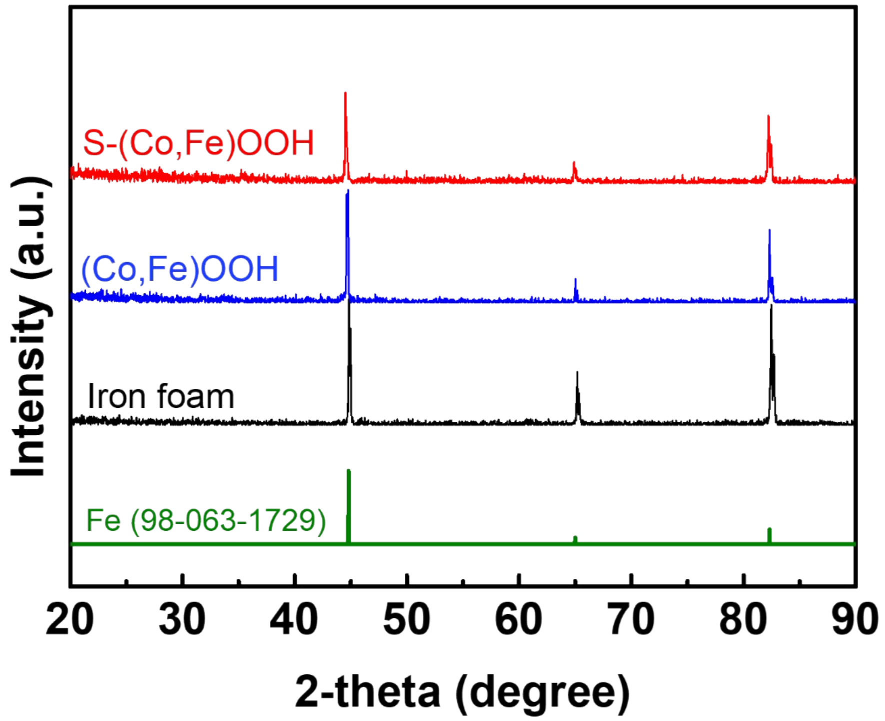

Figure S2. XRD patterns of Iron foam, (Co,Fe)OOH and S-(Co,Fe)OOH.

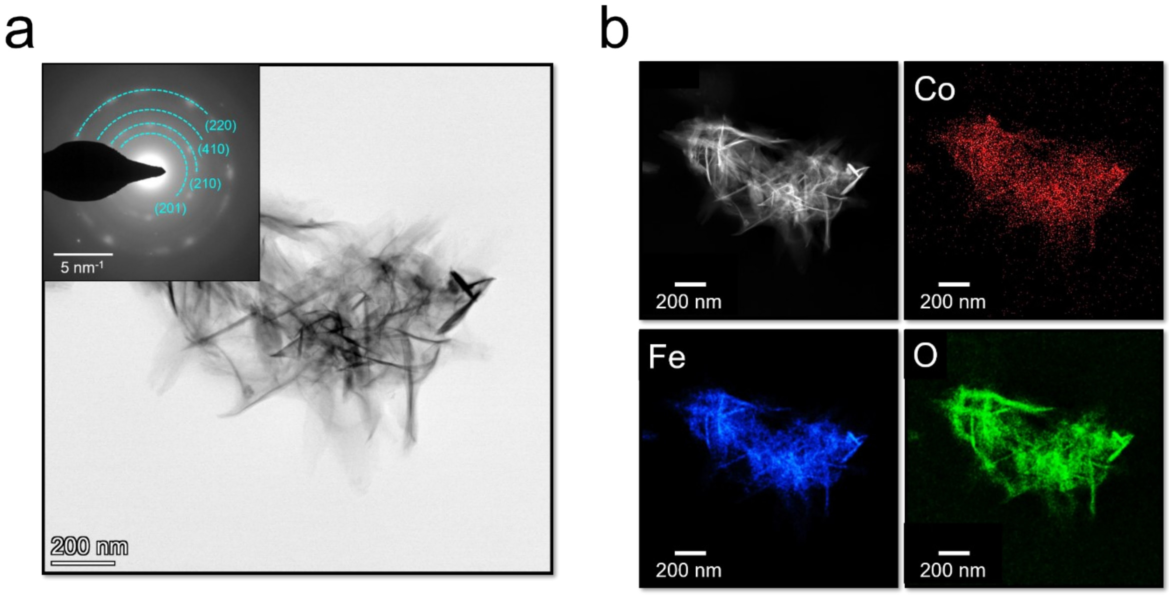

Figure S3. (a) High resolution transmission electron microscopy (HR-TEM) image of (Co,Fe)OOH with selected area electron diffraction (SAED) ring patterns. (b) TEM-EDS mapping images of (Co,Fe)OOH.

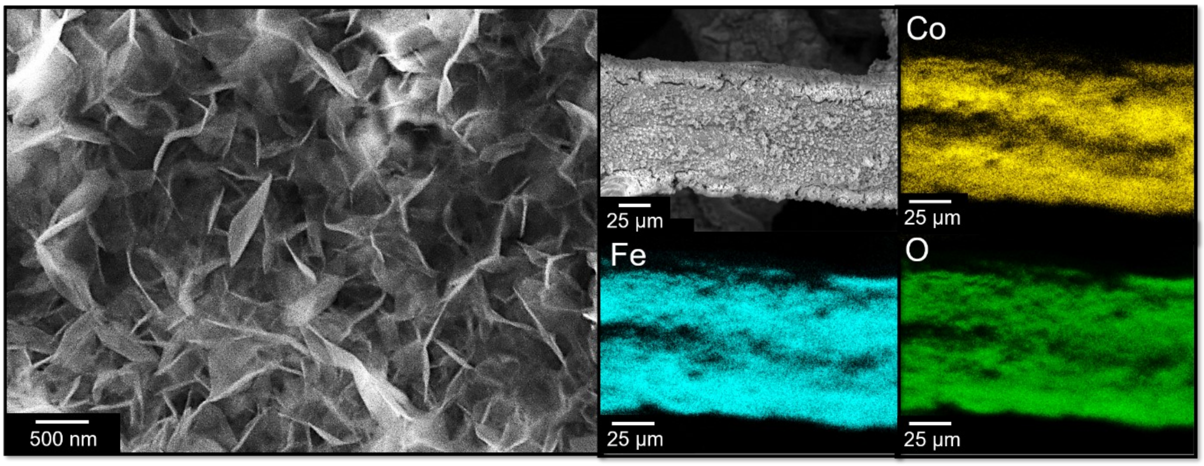

Figure S4. Scanning electron microscopy (SEM) image and Energy Dispersive Spectroscopy (EDS) mapping images of (Co,Fe)OOH .

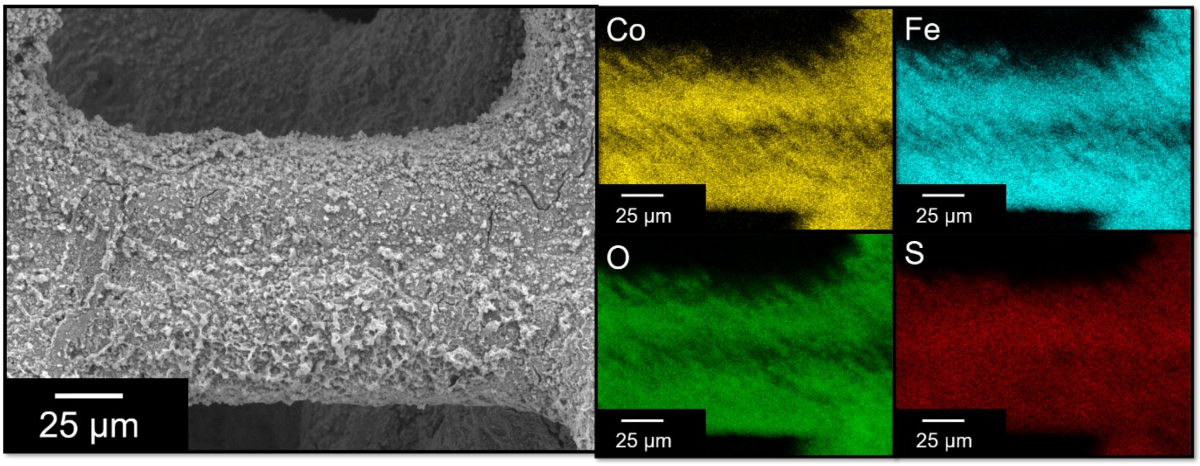

Figure S5. Energy Dispersive Spectroscopy (EDS) mapping images of S-(Co,Fe)OOH.

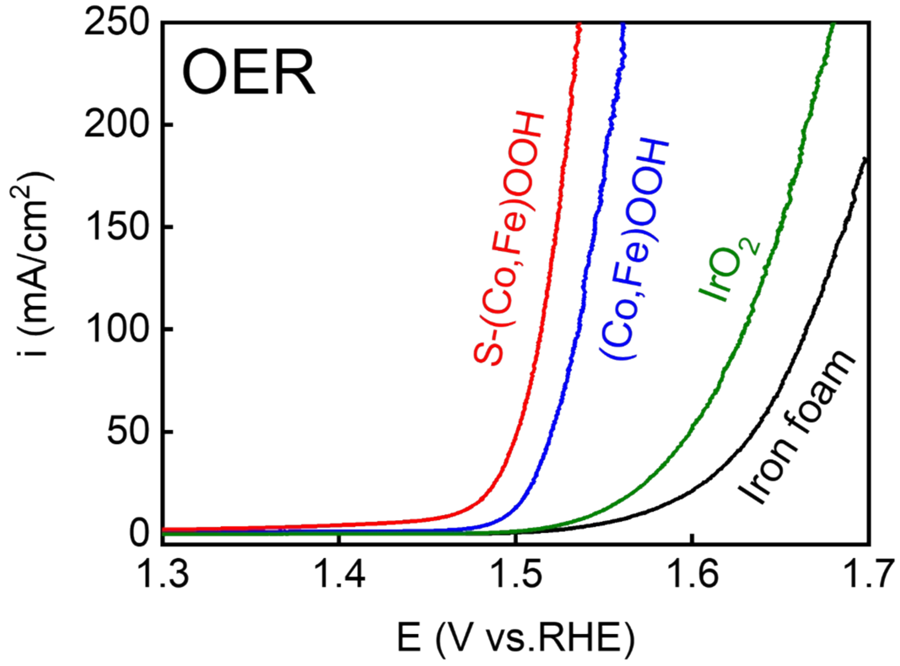

Figure S6. Forward sweeped polarization curves of Iron foam, IrO_2, (Co,Fe)OOH, and S-(Co,Fe)OOH for OER.

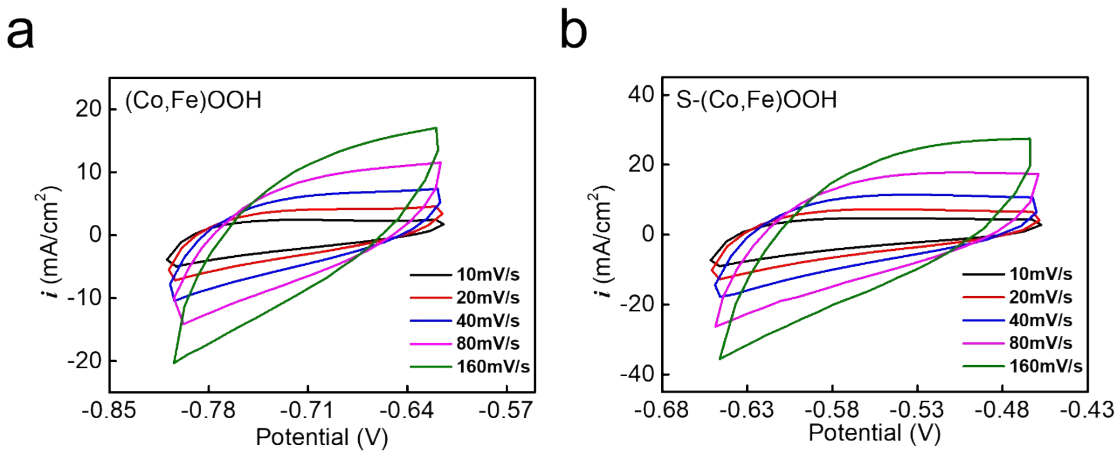

Figure S7. Cyclic voltammetry of (a) (Co,Fe)OOH and (b) S-(Co,Fe)OOH in non-faradaic region with different scan rates.

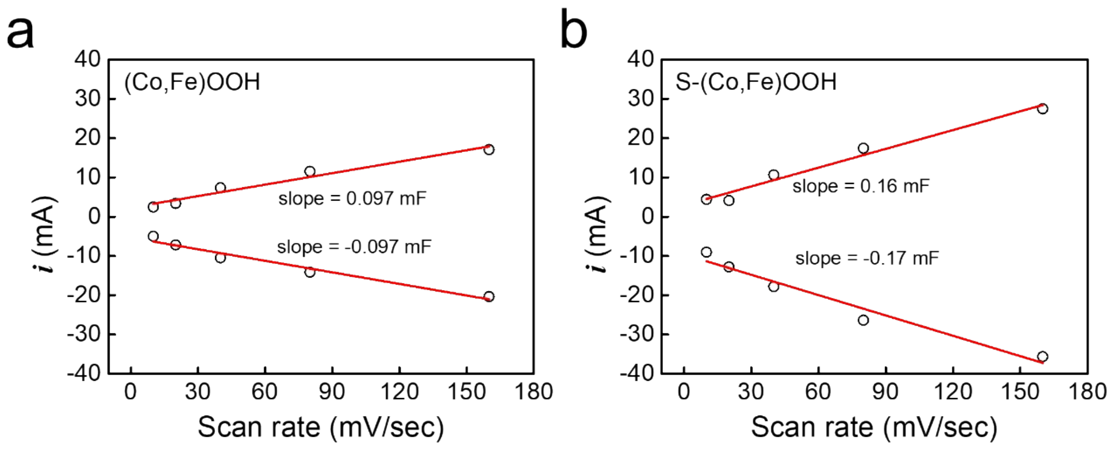

Figure S8. Double layer capacitance (C_{dl}) of (a) (Co,Fe)OOH and (b) S-(Co,Fe)OOH

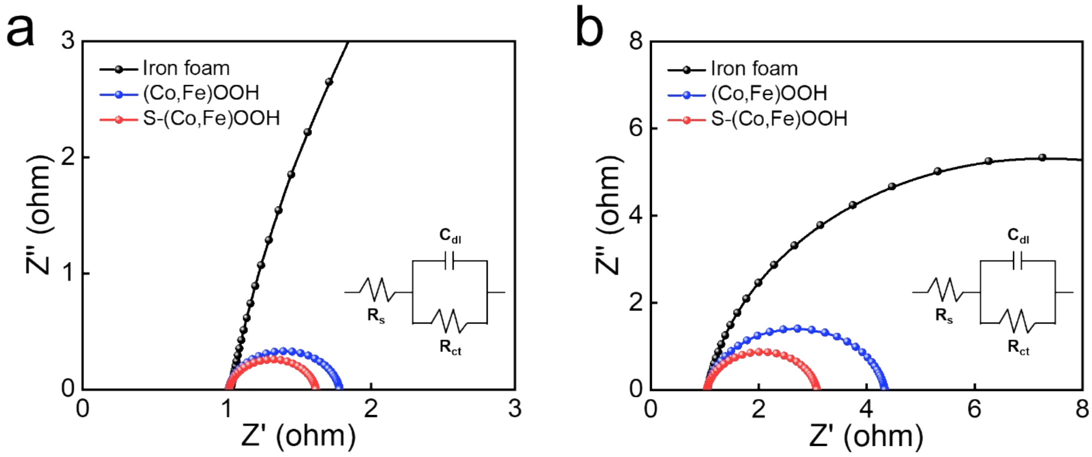

Figure S9. Electrochemical impedance spectroscopy (EIS) of Iron foam, (Co,Fe)OOH, and S-(Co,Fe)OOH for (a) OER at 1.53 V_{RHE} and (b) HER at -0.25 V_{RHE}.

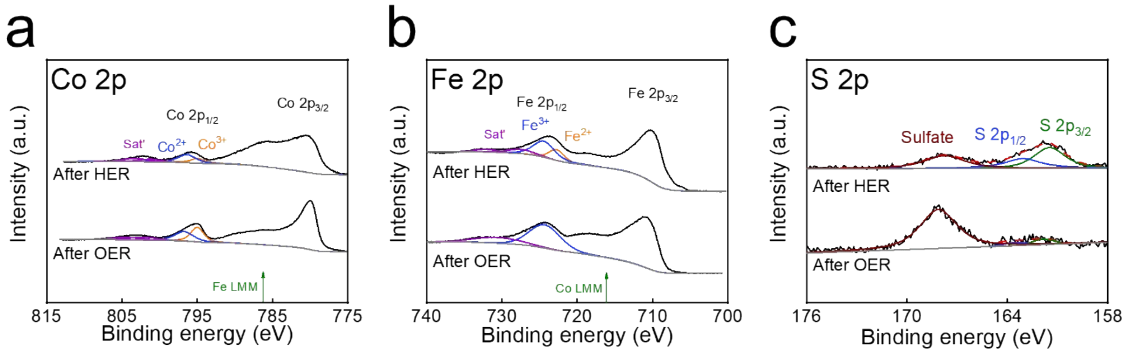

Figure S10. High-resolution XPS spectra of S-(Co,Fe)OOH after durability test. (a) Co 2p, (b) Fe 2p and (c) S 2p.

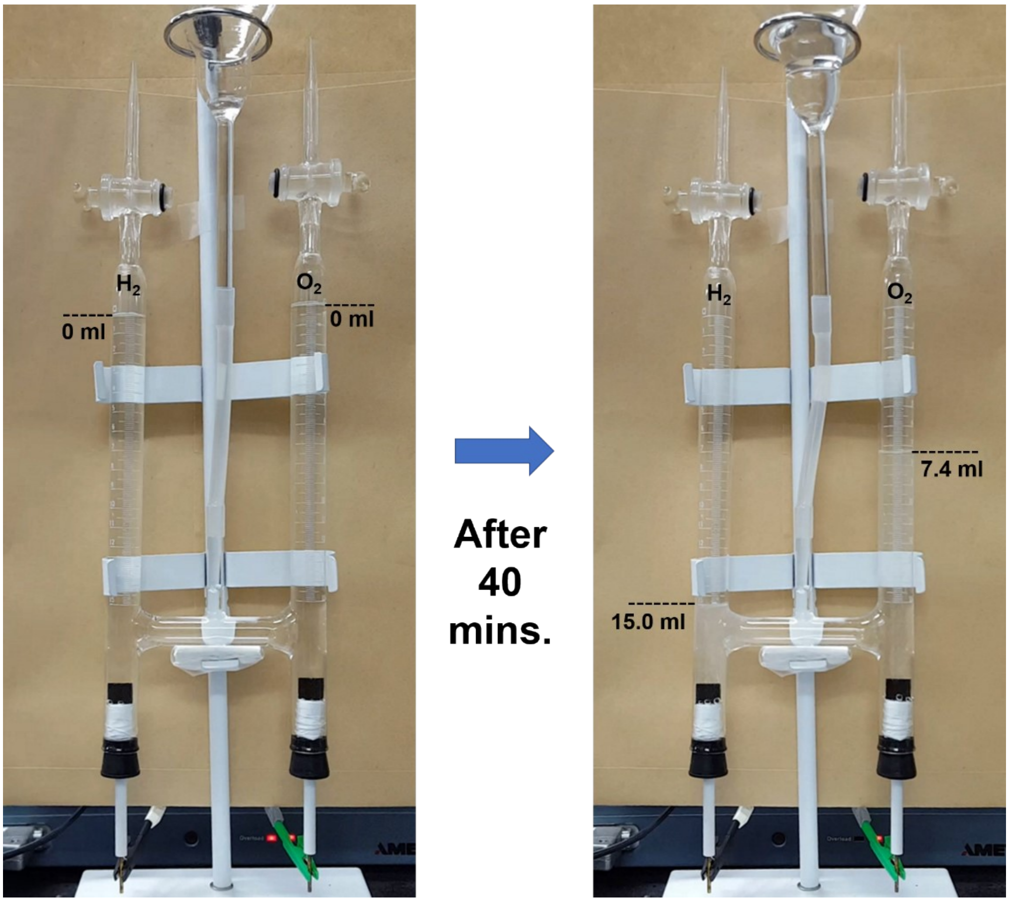

Figure S11. Faradaic efficiency measurement of S-(Co,Fe)OOH at 50 mA/cm^2 for 40 mins.

Table S1. Comparison of the electrocatalytic activity with recently reported transition metal-based catalyst for OER in 1 M KOH electrolyte.

Catalysts	η_j (mV)	j (mA/cm^2)	Tafel slope (mV/dec)	Electrolytes	Reference
S-(Co,Fe)OOH	240	10	39	1 M KOH	This work
	268	50			
	282	100			
Cr-doped FeNi-P/NCN	240	10	72.36	1 M KOH	1
	290	50			
$NiCo_2S_4$/NF	243	10	54.9	1 M KOH	2
	320	50			
Ni_2Fe_1O	244	10	39	1 M KOH	3
	273	50			
Ni−Fe-LDH−MoS_2	250	10	45	1 M KOH	4
$Mo_{51}Ni_{40}Fe_9$ nanobelts	257	10	51	1 M KOH	5
$NiCo_2S_4$	260	10	40	1 M KOH	6
$Fe_{0.5}Co_{0.5}P$	261	10	NA	1 M KOH	7
	281	50			
CeO_2/Co_3O_4 interface nanotubes	265	10	68.1	1 M KOH	8
CeO_x/CoS	269	10	50	1 M KOH	9
Ni_xS_y-N,S-doped carbon	270	10	68.9	1 M KOH	10
Ni_3FeN	280	10	46	1 M KOH	11
Fe-Ni_3S_2/FeNi	283	10	54	1 M KOH	12
	320	20			
$CoFe(OH)_{x-2}$/ Glassycarbon	293	10	67.4	1 M KOH	13
FeCoNi-ATNs/NF	295	10	52.7	1 M KOH	14
Co_9S_8@N-doped Carbon	302	10	67	1 M KOH	15
$CuCo_2S_4$	310	10	86	1 M KOH	16
HG-NiFe	313	10	39	1 M KOH	17
	350	50			
$CoMoS_3$ nanotube	320	10	NA	1 M KOH	18
	370	20			
NiFe-OH/NiFeP	323	10	77	1 M KOH	19
FeCo-P/C/Glassy carbon	360	10	58.4	1 M KOH	20

Table S2. Comparison of the electrocatalytic activity with recently reported transition metal-based catalyst for HER in 1 M KOH electrolyte.

Catalysts	η_j (mV)	j (mA/cm^2)	Tafel slope (mV/dec)	Electrolytes	Reference
S-(Co,Fe)OOH	186	-10	78	1 M KOH	This work
	236	-50			
	262	-100			
Ni−Fe-LDH−MoS_2	180	-10	77	1 M KOH	4
Ni-Fe-P nanocubes	180	-10	85.5	1 M KOH	21
Ni_3S_2/NF	189	-10	89.3	1 M KOH	22
Cr-doped FeNi– P/NCN	190	-10	68.51	1 M KOH	23
Co_3O_4- MTA	190	-20	98	1 M KOH	24
CoS_2 HNSs	193	-10	100	1 M KOH	25
NF@NiFe LDH	198	-10	130	1 M KOH	26
FeNi@NC/CNT	202	-10	113.7	1 M KOH	27
$Co_{0.75}Fe_{0.25}$@NC	202	-10	68	1 M KOH	28
Exfoliated NiFe LDH/defective	210	-10	110	1 M KOH	29
CoP@BCN-1	215	-10	52	1 M KOH	30
$Cu_{0.3}Co_{2.7}$P/NC	220	-10	122	1 M KOH	31
Co_2P	247	-10	103	1 M KOH	32
NiFeOF	253	-10	96	1 M KOH	33
Co/CoP-5	253	-10	73.8	1 M KOH	34
FeNi foam	299	-10	76.8	1 M KOH	35
NiFeP	355	-10	58.8	1 M KOH	36

References

1. Y. Wu, X. Tao, Y. Qing, H. Xu, F. Yang, S. Luo, C. Tian, M. Liu and X. Lu, *Advanced Materials*, 2019, **31**, 1900178.
2. Z. Kang, H. Guo, J. Wu, X. Sun, Z. Zhang, Q. Liao, S. Zhang, H. Si, P. Wu and L. Wang, *Advanced Functional Materials*, 2019, **29**, 1807031.
3. C. Dong, T. Kou, H. Gao, Z. Peng and Z. Zhang, *Advanced Energy Materials*, 2018, **8**, 1701347.
4. M. S. Islam, M. Kim, X. Jin, S. M. Oh, N.-S. Lee, H. Kim and S.-J. Hwang, *ACS Energy Letters*, 2018, **3**, 952-960.
5. X. Luo, Q. Shao, Y. Pi and X. Huang, *ACS Catalysis*, 2018, **9**, 1013-1018.
6. A. Sivanantham, P. Ganesan and S. Shanmugam, *Advanced Functional Materials*, 2016, **26**, 4661-4672.
7. Z. Wu, L. Huang, H. Liu and H. Wang, *ACS Catalysis*, 2019, **9**, 2956-2961.
8. B. Qiu, C. Wang, N. Zhang, L. Cai, Y. Xiong and Y. Chai, *ACS Catalysis*, 2019, **9**, 6484-6490.
9. H. Xu, J. Cao, C. Shan, B. Wang, P. Xi, W. Liu and Y. Tang, *Angewandte Chemie*, 2018, **130**, 8790-8794.
10. H. Yang, C. Wang, Y. Zhang and Q. Wang, *Small*, 2018, **14**, 1703273.
11. X. Jia, Y. Zhao, G. Chen, L. Shang, R. Shi, X. Kang, G. I. Waterhouse, L. Z. Wu, C. H. Tung and T. Zhang, *Advanced Energy Materials*, 2016, **6**.
12. C. Z. Yuan, Z. T. Sun, Y. F. Jiang, Z. K. Yang, N. Jiang, Z. W. Zhao, U. Y. Qazi, W. H. Zhang and A. W. Xu, *Small*, 2017, **13**, 1604161.
13. X. Yue, W. Ke, M. Xie, X. Shen, Z. Yan, Z. Ji, G. Zhu, K. Xu and H. Zhou, *Catalysis Science & Technology*, 2020, **10**, 215-221.
14. Q. Zhang, N. M. Bedford, J. Pan, X. Lu and R. Amal, *Advanced Energy Materials*, 2019, **9**, 1901312.
15. Z. Chen, R. Wu, M. Liu, Y. Liu, S. Xu, Y. Ha, Y. Guo, X. Yu, D. Sun and F. Fang, *Journal of Materials Chemistry A*, 2018, **6**, 10304-10312.
16. M. Chauhan, K. P. Reddy, C. S. Gopinath and S. Deka, *ACS Catalysis*, 2017, **7**, 5871-5879.
17. J. Wang, L. Gan, W. Zhang, Y. Peng, H. Yu, Q. Yan, X. Xia and X. Wang, *Science advances*, 2018, **4**, eaap7970.
18. J. Guo, X. Zhang, Y. Sun, L. Tang and X. Zhang, *Journal of Materials Chemistry A*, 2017, **5**, 11309-11315.
19. H. Liang, A. N. Gandi, C. Xia, M. N. Hedhili, D. H. Anjum, U. Schwingenschlögl and H. N. Alshareef, *ACS Energy Letters*, 2017, **2**, 1035-1042.
20. W. Hong, M. Kitta and Q. Xu, *Small Methods*, 2018, **2**, 1800214.
21. C. Xuan, J. Wang, W. Xia, Z. Peng, Z. Wu, W. Lei, K. Xia, H. L. Xin and D. Wang, *ACS Applied Materials & Interfaces*, 2017, **9**, 26134-26142.
22. L. Li, C. Sun, B. Shang, Q. Li, J. Lei, N. Li and F. Pan, *Journal of Materials Chemistry A*, 2019, **7**, 18003-18011.
23. Y. Wu, X. Tao, Y. Qing, H. Xu, F. Yang, S. Luo, C. Tian, M. Liu and X. Lu, *Advanced Materials*, 2019, **31**, 1900178.
24. Y. P. Zhu, T. Y. Ma, M. Jaroniec and S. Z. Qiao, *Angewandte Chemie International Edition*, 2017, **56**, 1324-1328.

25. X. Ma, W. Zhang, Y. Deng, C. Zhong, W. Hu and X. Han, *Nanoscale*, 2018, **10**, 4816-4824.
26. X. Wang, Y. Yang, L. Diao, Y. Tang, F. He, E. Liu, C. He, C. Shi, J. Li, J. Sha, S. Ji, P. Zhang, L. Ma and N. Zhao, *ACS Applied Materials & Interfaces*, 2018, **10**, 35145-35153.
27. X. Zhao, P. Pachfule, S. Li, J. R. J. Simke, J. Schmidt and A. Thomas, *Angewandte Chemie*, 2018, **130**, 9059-9064.
28. X. Feng, X. Bo and L. Guo, *Journal of Power Sources*, 2018, **389**, 249-259.
29. Y. Jia, L. Zhang, G. Gao, H. Chen, B. Wang, J. Zhou, M. T. Soo, M. Hong, X. Yan and G. Qian, *Advanced Materials*, 2017, **29**, 1700017.
30. H. Tabassum, W. Guo, W. Meng, A. Mahmood, R. Zhao, Q. Wang and R. Zou, *Adv. Energy Mater.*, 2017, **7**, 1601671.
31. J. Song, C. Zhu, B. Z. Xu, S. Fu, M. H. Engelhard, R. Ye, D. Du, S. P. Beckman and Y. Lin, *Advanced Energy Materials*, 2017, **7**, 1601555.
32. K. Xu, H. Ding, M. Zhang, M. Chen, Z. Hao, L. Zhang, C. Wu and Y. Xie, *Advanced Materials*, 2017, **29**, 1606980.
33. K. Liang, L. Guo, K. Marcus, S. Zhang, Z. Yang, D. E. Perea, L. Zhou, Y. Du and Y. Yang, *Acs Catalysis*, 2017, **7**, 8406-8412.
34. Y. Hao, Y. Xu, W. Liu and X. Sun, *Materials Horizons*, 2018, **5**, 108-115.
35. Y. Wu, F. Li, W. Chen, Q. Xiang, Y. Ma, H. Zhu, P. Tao, C. Song, W. Shang and T. Deng, *Advanced materials*, 2018, **30**, 1803151.
36. J. Lian, Y. Wu, H. Zhang, S. Gu, Z. Zeng and X. Ye, *International Journal of Hydrogen Energy*, 2018, **43**, 12929-12938.
